# Nutraceuticals in the Modulation of the Intestinal Microbiota: Current Status and Future Directions

**DOI:** 10.3389/fphar.2022.841782

**Published:** 2022-03-18

**Authors:** Enzo Spisni, Silvia Turroni, Patrizia Alvisi, Renato Spigarelli, Demetrio Azzinnari, Dario Ayala, Veronica Imbesi, Maria Chiara Valerii

**Affiliations:** ^1^ Department of Biological, Geological and Environmental Sciences, University of Bologna, Bologna, Italy; ^2^ Unit of Microbiome Science and Biotechnology, Department of Pharmacy and Biotechnology, University of Bologna, Bologna, Italy; ^3^ Pediatric Gastroenterology Unit, Maggiore Hospital, Bologna, Italy; ^4^ The Longevity Concept, Milano, Italy; ^5^ Department of Medical and Surgical Sciences, University of Bologna, Bologna, Italy

**Keywords:** microbiota, immunomodulation, probiotics, bacteria, prebiotic, postbiotic, essential oil, phytotherapy

## Abstract

Pharmaceutical interest in the human intestinal microbiota has increased considerably, because of the increasing number of studies linking the human intestinal microbial ecology to an increasing number of non-communicable diseases. Many efforts at modulating the gut microbiota have been made using probiotics, prebiotics and recently postbiotics. However, there are other, still little-explored opportunities from a pharmaceutical point of view, which appear promising to obtain modifications of the microbiota structure and functions. This review summarizes all *in vitro, in vivo* and clinical studies demonstrating the possibility to positively modulate the intestinal microbiota by using probiotics, prebiotics, postbiotics, essential oils, fungus and officinal plants. For the future, clinical studies investigating the ability to impact the intestinal microbiota especially by using fungus, officinal and aromatic plants or their extracts are required. This knowledge could lead to effective microbiome modulations that might support the pharmacological therapy of most non-communicable diseases in a near future.

## Introduction

### Gut Microorganisms and Human Health: A Complex Network

The gut microbiota (GM), i.e., the complex microbial community housed in our gastrointestinal tract, is undoubtedly a leading player in human physiology. Over the years, in fact, it has been shown to be responsible for numerous functions, from the barrier effect to the regulation of metabolism, to the modulation of the immune system, as well as the central nervous system (Sharon et al., 2016; Zheng et al., 2020; Barone et al., 2021). Among others, GM can indeed affect our energy balance and nutritional status, synthesizing essential vitamins (mainly those of group B) and making it possible to digest fibers by fermenting them into short-chain fatty acids (SCFAs, mainly acetate, propionate ad butyrate) (Flint et al., 2015). The latter are microbial metabolites considered essential for the maintenance of our metabolic, immunological, and neurological homeostasis, being involved among others in energy storage and expenditure, appetite control, strengthening of the integrity of the epithelial barrier, induction of interleukin (IL)10 and IL-18 production, and modulation of the synthesis and release of neuroactive substances (Koh et al., 2016; Makki et al., 2018). On the other hand, through the metabolism of dietary components, GM can also generate molecules with known harmful effects on human health, as exemplified by branched-chain fatty acids (related to insulin resistance, diabetes and inflammation), phenolic compounds (generally linked to poor cardiovascular outcomes), and trimethylamine (converted by the host into the proatherogenic trimethylamine-N-oxide) (see Turroni et al., 2018 for a review on the bioactive small molecules produced and/or contributed by GM and their effects along “the diet from the microbiome to the host axis”).

The vast majority of the aforementioned activities are attributed to the bacterial counterpart, by far the most studied and represented within the GM (with estimated over 10 trillion bacteria harboring a genetic potential hundreds of times greater than that of humans) (Sender et al., 2016), although more and more evidence is available on other GM fractions, such as the fungal (mycobiota) and the viral (virome) (Richard and Sokol, 2019; Shkoporov et al., 2019). In this regard, it should be remembered that microorganisms interact with each other in complex (often interkingdom) networks (Santus et al., 2021), whose ecological rules, in terms of positive (i.e., cooperation, commensalism, and cross-feeding), negative (competition, ammensalism, exploitation, and interference) and asymmetric interactions (exploitation, predation and parasitism) are still far from being understood (see Coyte and Rakoff-Nahoum, 2019 for a comprehensive review of microbe-microbe interactions within GM).

Such a complex microbial community is known to be shaped by a series of endogenous and exogenous variables, such as minimally genetics (Rothschild et al., 2018) and mostly exposome, i.e., “life-course environmental exposures (including lifestyle factors), from the prenatal period onwards” (Wild, 2005). Exposome mainly includes exposures related to personal behavior (diet, physical activity, drugs, etc.), occupational and those related to the built and outdoor environment (Zhang et al., 2019). In particular, as mentioned above, diet is widely recognized as one of the main variation drivers of GM, capable of influencing its composition and functionality, and cascading human physiology (Zmora et al., 2019). More recently, however, some authors have introduced the term “geographic effect” to describe the cumulative importance of personal and environmental exposures in driving the GM structure (He et al., 2018).

Based on the above, it is therefore not surprising that GM imbalances (i.e., dysbiosis) have been associated with a number of intestinal and extra-intestinal disorders, including metabolic, hepatic, immunological, respiratory, cardiovascular, neurological, psychiatric and oncological (Lynch and Pedersen, 2016). As will be detailed in the next paragraphs, the alterations in GM are generally featured by a reduction in diversity (a hallmark of health), the enrichment of opportunistic pathogens or pathobionts, and/or the depletion of beneficial microbes, primarily SCFA producers (Duvallet et al., 2017). This arrangement probably compromises the integrity of the epithelial barrier (i.e., leaky gut), with consequent translocation of microorganisms and elicitation of inflammatory states, both locally and systemically, potentially contributing to the onset of a plethora of non-communicable chronic diseases, including autoimmune ones (Gopalakrishnan et al., 2018; Camilleri, 2019). As recently discussed (Sonnenburg and Sonnenburg, 2019a,b), these maladaptive responses of GM are likely the result of a series of Westernization-related factors, including the consumption of industrialized and processed foods with low amounts of Microbiota-Accessible Carbohydrates (i.e., dietary fiber), the routine use of antibiotics and the increased sanitation, which have gradually depleted GM, depriving it of evolutionarily important microorganisms and interactions, thus leading to the establishment of the so-called microbiota insufficiency syndrome (Sonnenburg ED. and Sonnenburg JL., 2019).

### Dysbiosis and Non-communicable Diseases

**FIGURE 1:**
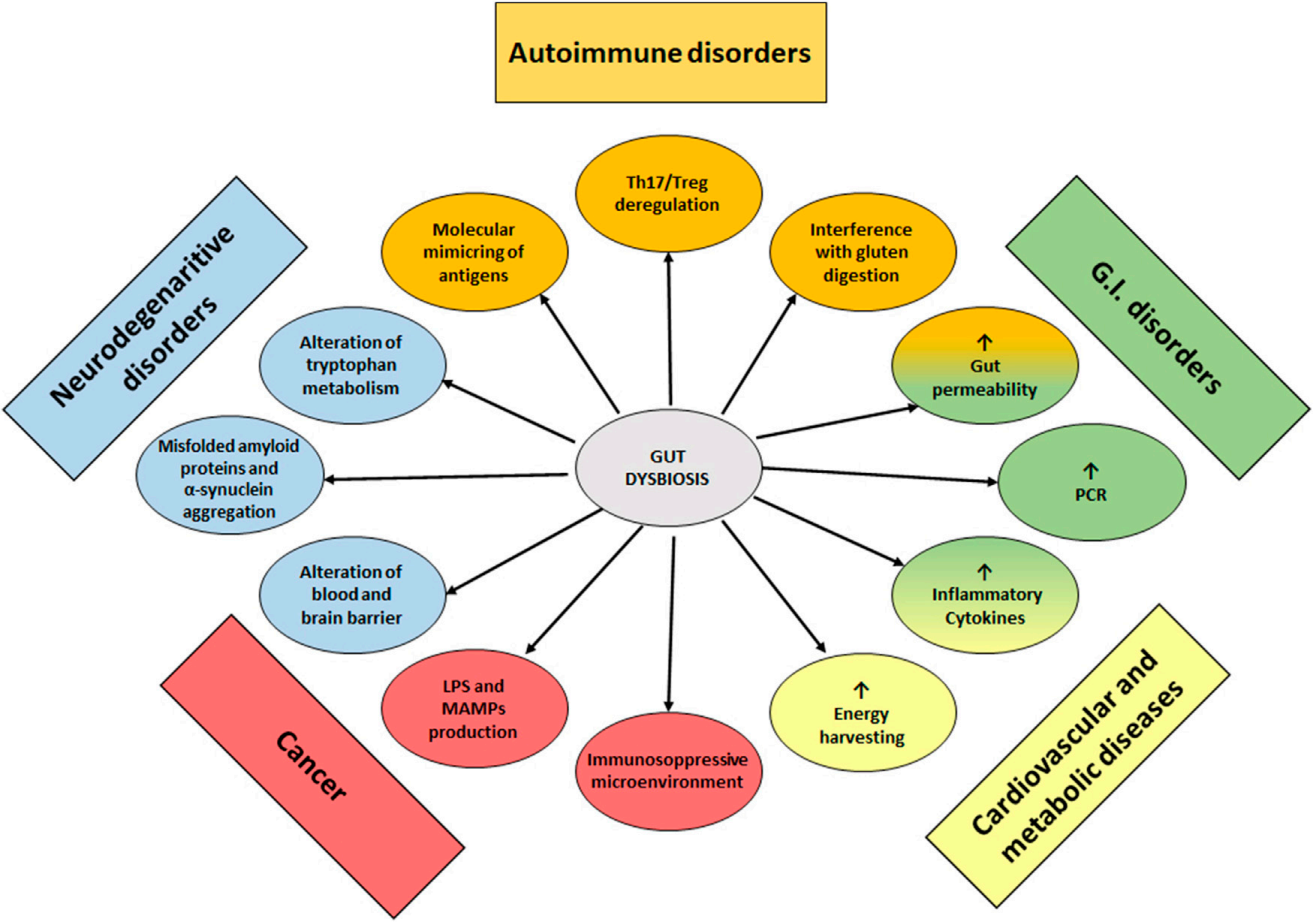
Dysbiosis of GM as a cause or contributing cause of non-communicable diseases.

The role of gut dysbiosis in the development of non-communicable disease has been summarized in Figure 1.

#### Dysbiosis and Autoimmune Disorders

Gut Associated Lymphoid Tissue (GALT) represents the immune system component in the gut and it is structured with specific subsets of immune cell populations, such as T and B lymphocytes, which protect the body from intracellular parasites and from perpetuating autoimmune responses (T helper 1 cells, Th1) or provide humoral response to fight extracellular organisms (T helper 2 cells, Th2). A balanced immune response involves the activity of other immune cell subsets such as T regulatory cells (Treg), which limit immune response preventing autoimmune responses, and T helper 17 (Th17), which inhibit Treg proliferation enhancing inflammatory response (Vangoitsenhoven and Cresci, 2020). Autoimmune Diseases (ADs) are disorders characterized by an immune response directed against self-antigenic proteins, which causes damage and loss of function of involved tissues. They are mainly classified as polygenic disorders (rheumatoid arthritis, systemic lupus erythematosus, type I diabetes) when several genes are involved in their pathogenesis, or monogenic disorders, when a single gene is associated with their onset (Liu et al., 2021). Although the presence of a genetic background predisposing to monogenic or polygenic ADs has been discovered, it is widely accepted that gut microbes and their metabolites may play a pivotal role in their onset and maintenance since they may affect intestinal permeability, with bacterial proteins translocating in the bloodstream, which in some cases mimic autoantigens and could interfere in the Th17/Treg cell balance (Atarashi et al., 2011). For example, segmented filamentous bacteria (Sczesnak et al., 2011; Goto et al., 2014) and bifidobacteria (Tanabe, 2013; Tan et al., 2016) can induce Th17 cell differentiation, while *Bacteroides fragilis* (Round et al., 2010), *Lactobacillus reuteri* and *Clostridium* cluster IV, XIVa and XVIII (Atarashi et al., 2011) have been demonstrated to promote the induction of colonic Tregs, the latter with a mechanism involving the production of SCFAs (Pryde et al., 2002). Type 1 Diabetes (T1D) has been associated with severe dysbiosis and increased intestinal permeability. In particular, an increase in *Bacteroides* and *Clostridium* and a decrease in bifidobacteria and lactobacilli have been found in these patients (Murri et al., 2013; Mejía-León et al., 2014; Pinto et al., 2017). Interestingly, *Bacteroides* overgrowth has been linked to Treg depletion and epithelial barrier impairment (Gianchecchi and Fierabracci, 2019). Moreover, in T1D patients, the increase in *Bacteroides* has been correlated to anti-islet cell autoantibodies (Henschel, et al., 2018). Hashimoto’s thyroiditis (HT) and Grave’s disease (GD) are the two main autoimmune disorders affecting the thyroid and are characterized by the presence of antibodies against thyreoperoxidase, thyroglobulin (in TH) and against thyroid-stimulating hormone (TSH) receptor in GD. In both HT and GD diseases, anti-gliadin, anti-transglutaminase and anti-*Saccharomyces cerevisiae* antibodies have been detected, and both thyroid autoimmune disorders are characterized by intestinal dysbiosis (Köhling et al., 2017). In HT patients, an increase in Prevotellaceae and Pasteurellaceae has been recorded and in particular, at the genus level, an increase in *Blautia*, *Roseburia*, *Ruminococcus*, *Romboutsia*, *Dorea*, *Fusicatenibacter* and *Eubacterium*. On the contrary, a decrease in Enterobacteriaceae, Veillonellaceae and the genera *Rikenella*, *Faecalibacterium, Bacteroides, Prevotella* and *Lachnoclostridium* has been highlighted (Ishaq et al., 2018; Zhao et al., 2018). It is more than plausible that a microbial pattern like this may affect Treg modulation and functions (Köhling et al., 2017). GM can modulate the synthesis of neurotransmitters, such as dopamine, which can inhibit TSH and modulate the hypothalamus-pituitary axis. So, an imbalance in GM could contribute to thyroid disorder development and maintenance with mechanisms involving microbial metabolic pathways (Zhao et al., 2018; Fröhlich and Wahl, 2019). Coeliac disease (CD) is an autoimmune disorder triggered by the interaction between GALT and undigested gluten peptides that translocate across the epithelial barrier into the lamina propria. An increased relative abundance of *Bacteroides*, *Prevotella* and *Escherichia*, and the concomitant reduction of bifidobacteria and lactobacilli have been supposed to contribute to the disease development by influencing the gluten peptide digestion, by stimulating dendritic cells and Tregs, and also by increasing intestinal permeability (Akobeng et al., 2020). Rheumatoid arthritis (RA) onset has been mainly associated with oral dysbiosis and periodontitis (Gianchecchi and Fierabracci, 2019). In the gut of RA patients, an overall decreased microbial diversity has been clearly evidenced (Chen et al., 2016; Picchianti-Diamanti et al., 2018), with a relatively greater abundance of *Prevotella copri* and a decrease in *Bacteroides* (Harmsen and and De Goffau, 2016; Schmidt et al., 2019). Interestingly, a study conducted by Pianta et al. (2017) evidenced epitopes in *P. copri*, which mimic N-acetyl glucosamine-6-sulfatase and filamin, 2 autoantigens highly expressed in RA patient synovia.

#### Dysbiosis in Gastrointestinal Disorders

Inflammatory Bowel Diseases (IBD) are chronic inflammatory disorders of the gastrointestinal tract with unknow etiology. Beyond a widely studied genetic background, lifestyle and diet appear to have a crucial impact on these pathologies. In fact, despite being a typical disease of Western countries, epidemiological studies reveal how their incidence is increasing in all populations that have adopted a Western lifestyle, characterized by the consumption of a diet enriched in saturated fats, sugars and processed foods. There is growing evidence that chronic inflammation in IBD is sustained by an inadequate response of the immune system to GM leading to unbalanced production of pro-inflammatory cytokines (Rizzello et al., 2019). GM in IBD patients has been extensively analyzed, with results often differing between studies, especially at lower taxonomic levels (Radhakrishnan et al., 2022). Nevertheless, there are some characteristics that have been confirmed by several studies that could explain the aberrant immune response driving chronic inflammation. A decrease in butyrate-producing species *Faecalibacterium prausnitzii* in IBD subjects, compared to healthy controls, has been evidenced and linked to Treg depletion (Sarrabayrouse et al., 2014; Khan et al., 2019; Akobeng et al., 2020). Moreover, an overall decrease in microbial diversity with a reduction of Firmicutes and an increase in Proteobacteria is often found in IBD patients and has been linked, at least in Crohn’s disease, to disease severity (Frank et al., 2007; Gevers et al., 2014; Maharshak et al., 2017). It is still debated if the dysbiosis can be linked to IBD onset or if it is a consequence of the disease. However, some clues are provided by studies in IBD patients with anal-ileus pouch. The pouch is a transitional tissue which, after surgery, is colonized *de novo* by GM (Falk et al., 2007; Kohyama et al., 2009). In a prospective study, a decrease in microbial diversity was observed in fecal samples analyzed before surgery in patients developing pouch inflammation, compared to those who maintained a normal (non-inflamed) pouch (Maharshak et al., 2017). Moreover, an increase in *Ruminococcus* and *Clostridium perfringens* was found in a similar study, associated with a reduction in *Blautia* and *Roseburia* (Machiels et al., 2017). The efficacy of antibiotics and some probiotics for the management of inflammatory flares seems to confirm the strong impact that, beyond a well-established genetic background, GM composition and functions may have on these diseases (Dalal et al., 2018). Irritable Bowel Syndrome (IBS) is a functional disorder characterized by abdominal pain, bloating and an altered intestinal habit that defines four main IBS subtypes: diarrhea-predominant (IBS-D), constipation-predominant (IBS-C), mixed diarrhea and constipation (IBS-M) and non-classifiable IBS symptoms (IBS-U) (Hellström and Benno, 2019). GM analysis in these patients revealed a dysbiosis characterized by loss of diversity compared to the healthy population. Again, the results from various studies are often conflicting. For example, the ratio Firmicutes/Bacteroidetes was found to be both higher and lower in different studies (Jalanka-Tuovinen et al., 2014; Pozuelo et al., 2015). Conflicting results were also obtained on the abundance of SCFA producers (Rodiño Janeiro et al., 2018). These differences may be due to the different analytic techniques, but also to the different clinical features that characterize the IBS subtypes and that should be considered in data analysis (Rodiño Janeiro et al., 2018). Overall, the most consistent data in IBS describe increased proportions of Proteobacteria and Firmicutes members, such as *Veillonella* and *Ruminococcus* (Malinen et al., 2005; Tana et al., 2010; Saulnier et al., 2011), and a decrease in *Lactobacillus*, *Bifidobacterium, Faecalibacterium* and methanogens (Malinen et al., 2005; Rajilic´-Stojanovic´ et al., 2011; Zhuang et al., 2017; Zhuang et al., 2017).

#### Dysbiosis in Metabolic and Cardiovascular Diseases

GM regulates the host energy balance by various mechanisms, e.g., by inducing monosaccharide uptake from the gut and hepatic production of triglycerides, by regulating nutrient absorption and the expression of adipokines, such as the fasting-induced adipose factor, which are involved in peripheral fat storage, as clearly shown by studies on germ-free mice (Bäckhed et al., 2004; Tilg et al., 2020). GM is responsible for the production of SCFAs and monosaccharides from indigestible carbohydrates in the diet, such as fiber (Flint et al., 2015). In metabolic disorders such as obesity and type 2 diabetes, which are strictly correlated to cardiovascular outcomes, intestinal dysbiosis triggers or contributes to the exacerbation and chronicization of these conditions by impairing metabolic pathways and inducing chronic low-grade inflammation that is a typical feature of these diseases (Angelakis et al., 2012; Cotillard et al., 2013). Less microbial diversity has been found in obese subjects, with an overrepresentation of proinflammatory taxa, such as *Ruminococcus* and *Bacteroides*, and a decrease in anti-inflammatory species such as *F. prausnitzii* (Cotillard et al., 2013). Moreover, in subjects with metabolic syndrome there is a greater abundance of SCFA producers, with an increased ability to extract calories from low-energy foods (Marzullo et al., 2020). In type 2 diabetes subjects, GM analysis showed a decrease in *Roseburia intestinalis* and *F. prausnitzii*, associated with high intestinal transport (absorption) of sugars (Qin et al., 2012). A common feature in patients with metabolic disorders is the higher proportion of gram-negative bacteria associated with higher levels of lipopolysaccharide (LPS), a bacterial toxin responsible for increased intestinal permeability and endotoxemia, which leads to systemic inflammation mediated by proinflammatory cytokines (Cani et al., 2007; Qin et al., 2012). This feature may be considered the link between metabolic disorders and cardiovascular diseases such as atherosclerosis, which damages blood vessels predisposing to arterial plaque formations (Novakovic et al., 2020). On the other hand, into the atherosclerotic plaques, *Crysenomonas*, *Helicobacter*, *Anaeroglobus, Clostridium, Eubacterium,* and *Roseburia* have been detected (Koren et al., 2011), while in the gut the overrepresentation of *Lactobacillus, Bacteroides, Collinsella,* and *Streptococcus* has been suggested as a diagnostic marker in patients suffering from cardiovascular disease (Emoto et al., 2017). Together with low gut diversity, some bacterial genera such as *Lactobacillus, Oscillibacter, Faecalibacterium* and *Ruminococcus* showed some correlations with a higher level of C-reactive protein, which is considered a marker of inflammation and cardiovascular disease (Kazemian et al., 2020). From the metabolic point of view, some species belonging to Proteobacteria and Firmicutes have been associated with the synthesis of trimethylamine (TMA) and its derivative trimethylamine N-oxide (TMAO), which is considered a risk factor for myocardial infarction and stroke (Tang et al., 2013; Liu et al., 2015; Hu et al., 2017).

#### Dysbiosis in Cancer

GM involvement in carcinogenesis is mainly linked to the release of some chemical mediators called microorganism-associated molecular patterns (MAMPS), which can enhance tumor progression or cause an impairment of the immune system (Schwabe and Jobin, 2013). In animal models of hepatocellular carcinoma as well as in pancreatic cancer, LPS released from GM is able to activate Toll-like receptor 4 (TLR4) and negatively modulate immune responses (Dapito et al., 2012; Ochi et al., 2012). Furthermore, several studies showed a typical GM signature in cancers, such as colorectal cancer (CRC) in which a particular strain of *Fusobacterium nucleatum* appears to be involved in tumor initiation and progression by inducing the local release of pro-inflammatory cytokines, such as IL-6, IL-8 and TNF-α, thus developing an immunosuppressive tumor-favoring microenvironment (Shang and Liu, 2018). In non-small cell lung cancer, localized depletion of butyrate producers such as *F. prausnitzii, Clostridium leptum*, *Clostridium* cluster I, *Ruminococcus* spp., *Clostridium* cluster XIVa, and *Roseburia* spp., is supposed to deregulate Treg differentiation and immune responses against the cancer cells (Cheng et al., 2019; Gui et al., 2020). Acute lymphoblastic leukemia is characterized by increased abundance of Enterococcaceae, Porphyromonadaceae and other Bacteroidetes members, and by depletion in *Blautia*, Erysipelotrichales, Lachnospiraceae and Clostridiales members in the GM, while in acute myeloid leukemia, an abundance of Staphylococcaceae and Streptococcaceae has been found (Dutta and Lim, 2020).

#### Dysbiosis and Neurodegeneration

There are different ways by which GM contributes to the physiological modulation of the central nervous system (CNS). For example, commensal bacteria such as *Escherichia* (Lee et al., 2020), *Streptococcus* (Macrì et al., 2018) and *Enterococcus* may contribute to the regulation of dopamine levels, while *Bifidobacterium* and *Lactobacillus* can produce gamma-aminobutyric acid, which is also involved in dendritic and T cell differentiation (Powell et al., 2017). The inflammatory component associated with neurodegeneration and the contribution of GM in the regulation of neurotransmission make it plausible to associate psychiatric or neurodegenerative disorders such as Alzheimer Disease (AD), Parkinson Diseases (PD), Multiple Sclerosis (MS), Amyotrophic lateral sclerosis (ALS) and Huntington’s disease with GM alterations (Uniyal et al., 2021). Interestingly, epidemiological data suggest that diet could play a preventive or delaying role in prion and prion-like neurodegenerative diseases, and diet is a major driver of GM variation (Toni et al., 2017). Nevertheless, to date, the most reliable data on the connection between neurodegeneration and GM come from studies on AD and PD. In AD, an altered GM appears to be linked to LPS production, alterations of the blood-brain barrier and deregulation of Treg/Th2 responses, which promote AD development and fibrillogenesis of β-Amiloid (Li et al., 2017; Tsunoda 2017). Instead, amyloid proteins may be released by GM components, and it has been observed that *Escherichia coli*, *Salmonella* Typhimurium*, Bacillus subtilis, Mycobacterium tuberculosis, Salmonella enterica,* and *Staphylococcus aureus* are all bacteria capable of generating functional amyloid, contributing to accumulation of proteinaceous misfolded particles, oligomers and fibrils (Jiang et al., 2017; Fang et al., 2020).

In PD patients, impairment of the motor system has been associated with the presence of non-motor symptoms, linked to autonomic dysfunctions, now recognized as a part of this disease. Among these autonomic dysfunctions, intestinal constipation can arise even 20 years before the onset of motor symptoms and could be linked to GM alterations (Mertsalmi et al., 2017). An increase in the relative abundance of Bifidobacteriaceae, Lactobacillaceae and Verrucomicrobiaceae has been observed (Fang et al., 2020) and the increase in Enterobacteriaceae has been clearly associated with postural instability (Sampson et al., 2016). Furthermore, the decrease in *Prevotella* has been associated with reduced mucin synthesis and increased gut permeability in PD patients (Sampson et al., 2016). These modifications also correlate with alterations in the microbial metabolism of tryptophan and beta-glucuronides (Bedarf et al., 2017), involved in neurotransmitter biosynthesis. In addition to this, GM dysbiosis could be involved in PD development by enhancing alpha-synuclein aggregation in the intestinal submucosa neurons, which may propagate to the CNS via the vagal nerve route (Fang et al., 2020; Uniyal et al., 2021).

The major GM alterations observed in non-communicable diseases are summarized in Table 1.

**TABLE 1:** Main GM modifications (referred to as taxa) observed in several non-communicable diseases.

	Increased taxa	Decreased taxa
Autoimmune disorders		
Type 1 Diabetes	*Bacteroides, Clostridium*	Bifidobacteria, lactobacilli
Hashimoto’s Thyroiditis	Prevotellaceae*,* Pasteurellaceae	Enterobacteriaceae, Veillonellaceae
	*Blautia*, *Roseburia*, *Ruminococcus*, *Romboutsia*, *Dorea*, *Fusicatenibacter*, *Eubacterium*	*Rikenella*, *Faecalibacterium, Bacteroides, Prevotella, Lachnoclostridium*
Coeliac Disease	*Bacteroides*, *Prevotella, Escherichia*	Bifidobacteria, lactobacilli
Rheumatoid Arthritis	*Prevotella copri*	*Bacteroides*
Gastrointestinal Disorders		
Inflammatory Bowel Diseases	Proteobacteria	Firmicutes*, Faecalibacterium prausnitzii*
Pouchitis	*Ruminococcus, Clostridium perfringens*	*Blautia, Roseburia*
Irritable Bowel Syndrome	Proteobacteria, Firmicutes (*Veillonella, Ruminococcus*)	*Lactobacillus*, *Bifidobacterium, Faecalibacterium,* methanogens
Metabolic and Cardiovascular Diseases		
Obesity	*Ruminococcus, Bacteroides*	*F. prausnitzii*
Metabolic Syndrome	*SCFA producers*	
Type 2 Diabetes		*Roseburia intestinalis* and *F. prausnitzii*
Cardiovascular Diseases	*Lactobacillus, Bacteroides, Collinsella, Streptococcus, Lactobacillus, Oscillibacter, Faecalibacterium, Ruminococcus,* TMA producers*	
Cancer		
Colorectal Cancer	*Fusobacterium nucleatum*	
Non-Small Cell Lung Cancer		*F. prausnitzii, Clostridium leptum*, *Clostridium* cluster I, *Ruminococcus* spp., *Clostridium* cluster XIVa, *Roseburia* spp.
Acute Lymphoblastic Leukemia	Enterococcaceae, Porphyromonadaceae	*Blautia*, Erysipelotrichales, Lachnospiraceae, Clostridiales
Myeloid Leukemia	Staphylococcaceae, Streptococcaceae	
Neurodegenerative Disorders		
Alzheimer’s Disease	Escherichia coli*, *Salmonella* Typhimurium*, Bacillus subtilis*, Mycobacterium tuberculosis*, Salmonella enterica*, Staphylococcus aureus*	
Parkinson’s Disease	Bifidobacteriaceae, Lactobacillaceae*,* Verrucomicrobiaceae*,* Enterobacteriaceae	*Prevotella*

### Prebiotics, Probiotics and Postbiotics for the Microbiota Modulation (Effectiveness and Limits)

Due to its crucial role in human pathophysiology, as detailed above, GM is increasingly considered a therapeutic target in multiple clinical settings, and its modulation is strongly believed to represent an important adjunct to current intervention strategies. Historically, such modulation can be achieved through the use of prebiotics or probiotics (or their combination, synbiotics) or, more recently, postbiotics. Below, for each of these manipulation tools, the available evidence and current limitations will be briefly discussed.

Prebiotics, i.e., “a substrate that is selectively utilized by host microorganisms conferring a health benefit” (Gibson et al., 2017), typically include dietary fiber and oligosaccharides, such as fructo-oligosaccharides, inulin and galacto-oligosaccharides. Human milk oligosaccharides, conjugated linoleic acids, polyunsaturated fatty acids, phenolics and phytochemicals are still considered candidate, i.e., molecules whose prebiotic potential has been demonstrated *in vitro* or in animal models but for which human evidence is still insufficient. The health effects of prebiotics are innumerable and extend far beyond the gastrointestinal system, including for example immune responses, metabolism, skin, bones, central nervous system, etc. (see Table 1 from Gibson et al., 2017). These effects are mediated by GM fermentation, therefore by the production of metabolites, mainly SCFAs, which derive from the establishment of complex syntrophic networks, mostly involving members of the dominant families Lachnospiraceae, Ruminococcaceae and Bacteroidaceae (Candela et al., 2010). For example, it has recently been shown that inulin-type fructans are not only bifidogenic, but also induce specific changes in human GM, namely increase in bifidobacteria (as primary degraders) and *Alistipes* (which benefits from degradation by *Bifidobacterium* through cross-feeding), and decrease in *Bilophila*, due to reduced pH and altered conjugation ratios of bile acids (Vandeputte et al., 2017). Despite such evidence, it is still hard to say which prebiotic to administer to achieve certain effects in GM, at what dose and whether this dose will actually be tolerable. Some interesting insights in this direction have recently been provided by Deehan et al. (2020). According to the authors, chemically modified resistant starches with small structural differences, i.e., crystalline maize resistant starch and cross-linked tapioca resistant starch, are capable of inducing divergent and highly specific effects on GM, with enrichment in *Eubacterium rectale* or *Parabacteroides distasonis*, that direct changes in the output of butyrate and propionate, respectively. Notably, dominant effects were consistent within treatment groups and dose-dependent with a plateau at 35 g. While it is still impossible to predict with certainty the changes in GM and human physiology, this study opens the door to the fascinating possibility of developing carbohydrates designed *ad hoc* for a targeted, systematic, and precision manipulation of GM and its metabolic functions relevant to health.

Based on consensus panel recommendations in 2014 (Hill et al., 2014), probiotics, *i.e.*, “live microorganisms that, when administered in adequate amounts, confer a health benefit on the host”, include microbial species that have been shown in properly controlled studies to confer benefits to health, as well as potential new commensals and consortia, comprising defined strains from human samples, for which adequate evidence of safety and efficacy is available. In contrast, live cultures, traditionally associated with fermented foods and for which there is no evidence of a health benefit, as well as undefined, fecal microbiota transplants must be kept outside the probiotic framework. To date, probiotics are known to exert their health benefits through a series of mechanistic interactions with the host and the GM, concerning the metabolism of nutrients (with improvement of lactose tolerance), the direct and indirect pathogen antagonism, improved barrier function, immunomodulation, the analgesic effect on visceral pain, the change in signaling to the nervous system and, of course, the alteration of GM (see Suez et al., 2019 for a review on the pros, cons and many unknowns of probiotics). These effects may depend on contact and/or be mediated by surface (e.g., lipoteichoic acid, exopolysaccharides and cell surface appendages) and secreted (e.g., SCFAs, bacteriocins, etc.) molecules. With particular regard to GM, probiotics are able to transiently integrate into GM and, once they become part of the transient microbiota, they can influence its composition and activity in many ways, including stimulation of the resident community by trophic interaction (through metabolites, growth factors, carbohydrate metabolism, mucin degradation), reduction/inhibition of pathogens through alteration of microbial fitness (pH decrease, niche competition, bacteriocins), and indirect impact via host through changes in the intestinal environment (mucin production, increase of sIgA and defensins) (Derrien and van Hylckama Vlieg, 2015). It should be remembered that these mechanisms are not shared by all probiotics known to date and that, in particular, the production of specific bioactives and the immunological, endocrinological and neurological effects tend to be strain-specific (Hill et al., 2014). Furthermore, it must be said that sometimes conflicting data were obtained, partly related to the heterogeneity of probiotic agents, dosage, duration and mode of administration used in the different studies, but also to other host variables that may mask the true probiotic impact, especially the habitual diet. In this regard, fortunately, there is a growing awareness that one size does not fit all but that baseline host and GM features should be taken into account for a precision, personalized therapy (Suez et al., 2019). As a proof of concept, in 2018 some researchers demonstrated that GM profiles may be resistant vs. permissive to probiotics colonization (Zmora et al., 2018), and especially that probiotic interventions, if not tailored, may not only be ineffective but also not entirely risk-free (Suez et al., 2018). Future directions in this field should therefore include changes at multiple levels, such as strain-level resolution of clinical and mechanistic studies, adequate sample size, definition of highly valid and reliable endpoints, reporting of adverse effects, long-term safety assessment and the inclusion of novel candidate microorganisms with suggested health benefits from recent microbiome research, i.e., next-generation probiotics or live biotherapeutics (O’Toole et al., 2017). Regarding the latter, unfortunately most are still at a very early stage of mechanistic investigation, with the exception of *Akkermansia muciniphila*, a mucin degrader proposed and tested for the treatment of obesity and related complications, and recently approved by EFSA in pasteurized form (EFSA Panel on Nutrition, Novel Foods and Food Allergens (NDA) et al., 2021). In particular, in a proof-of-concept exploratory study in overweight and obese human volunteers, pasteurized *A. muciniphila* improved insulin sensitivity and reduced insulinemia, plasma total cholesterol, body weight, fat mass and hip circumference, without affecting the overall structure of the GM (Depommier et al., 2019).

As an alternative to probiotics, with a longer shelf-life and increased safety especially for immunocompromised individuals, the use of non-viable microorganisms and/or their components has been proposed. These are collectively referred to as postbiotics, i.e., “preparation of inanimate microorganisms and/or their components that confers a health benefit on the host”, and precisely include non-viable cells with or without metabolites or cell components, capable of conferring beneficial effects to the host directly or indirectly (e.g., enzymes, peptides, teichoic acids, peptidoglycan-derived muropeptides, polysaccharides, cell surface proteins and organic acids) (Salminen et al., 2021). Postbiotics would exert their actions through five postulated mechanisms, namely modulation of GM (e.g., through the antimicrobial activity of bacteriocins or lactic acid, by carrying quorum sensing and quorum quenching molecules, by providing carbon sources and also through competition with pathogens if adhesins remain intact after processing), strengthening of barrier function, modulation of local and systemic immune responses, modulation of systemic metabolic responses, and systemic signaling through the nervous system. Notwithstanding the need for high-quality randomized placebo-controlled (or alternatively, active agent-controlled) trials, the available evidence suggests that postbiotics may prove effective in adults as new antimicrobials, targeted anti-inflammatory and immunoregulatory agents, and signaling molecules, while there is only limited evidence on the health benefits of including them in infant formulas (see Table 2 and 3 from Salminen et al., 2021). Importantly, several issues must be considered for a preparation to be qualified as such, such as detailed description of the starting material (including the molecular characterization of progenitor microorganisms), the means of inactivation (and confirmation that it has occurred) and assurance of safety in the targeted host for the intended use. Only careful control of these parameters will allow reliable and repeatable research for the integrated use of postbiotics in medical and pharmaceutical applications.

### Fungus and Plant Extracts for Microbiota Improvement

The modulation of GM and, consequently, of the gut immune system is a key aspect to preserve the correct physiology of the gastrointestinal tract. GM alterations are continuously reflected at the immune level and therefore may become systemic. Consequently, the action of fungi and medicinal plants is exerted on the gastrointestinal system through immunomodulating, antioxidant and protective properties on GM. The protection of the intestinal biofilm and barrier, structures in which the GM actively and directly participates, also fall within the therapeutic actions of fungus and therapeutic plants. These effects on the intestinal barrier and on the gastrointestinal system can have, as we have seen, multiple systemic consequences. Several medicinal plants and fungi are described in the scientific literature as being able to act positively on various acute and chronic inflammatory disorders of the gastrointestinal system, most of these are also part of the medical tradition of one or more regions of the world. Despite this, the actions on the GM have been studied in preclinical and controlled clinical studies for only a few medicinal plants and fungi. Sometimes, studies in the literature support the possible therapeutic use of some of these fungi and plants only in the modulation of intestinal inflammation. Although it is evident that the inflammatory component and the alteration of the GM are associated in almost all pathologies of the gastrointestinal system, it is not possible to deduce from the effects on inflammation which kind of modulations in the GM actually occurred.

#### Microbiota-Modulating Fungi

*Hericium erinaceus* is the most used mushroom for all gastrointestinal disorders. It is an edible mushroom, which has a long history of use in traditional Chinese medicine for the protection of mucous membranes, gastric ulcers, acute and chronic gastritis and nervous degeneration (Khan et al., 2013; Friedman, 2015; Thongbai et al., 2015). The parts of the fungus used are the fruiting body and/or the mycelium in aqueous, hydroalcoholic or alcoholic extracts titrated and standardized in one or more of the following components: polysaccharides, beta-glucans (with antibacterial and anti-inflammatory action), alpha -glucans, diterpenes and triterpenes or polyphenols (He et al., 2017). Although the most studied activities of this fungus concern its immunomodulatory effects on the gut, its prebiotic activities have aroused much interest (Sheng et al., 2017). A single protein, called HEP3, isolated from *H. erinaceus* and administered to rats, treated with trinitrobenzenesulfonic acid (TNBS) to induce experimental colitis, was able to restore microbiota diversity in a relatively short time. Treatment with this single *H. erinaceus* protein increased the amounts of Actinobacteria and Tenericutes, reduced those of Bacteroidetes and Firmicutes, and was able to restore a biodiverse and healthy ecological structure (Diling et al., 2017). The efficacy of HEP3 in positively modulating the GM has also been confirmed in other animal models of colitis (Shao et al., 2019). Crude extracts of *H. erinaceus* were also tested on animal models of colitis. The results indicate that the formulations used (polysaccharide extract, alcoholic extract or whole extract) were able to positively modulate the GM, but while the polysaccharide extract appeared to play an important prebiotic role, the alcoholic extract and the whole extract showed important bactericidal effects (Diling et al., 2017). Similar results were obtained in a mouse model of sodium sulfate dextran-induced colitis (DSS). Treatment with DSS resulted in an increase in the relative abundance of Verrucomicrobia and Actinobacteria and a decrease in the amount of Bacteroidetes in fecal samples, compared to the control group. Treatment of colitic mice with dry extract of the fermented mycelium of *H. erinaceus* reversed most of the changes, including the increased levels of *A. muciniphila*. Taken together, these results showed that *H. erinaceus* effectively modulates the GM of colitic animals, restoring a microbial composition similar to that of healthy rodents (Ren et al., 2018).

*Inonotus obliquus* commonly known as Chaga is a parasitic fungus mainly of Birch trees (Betulaceae family) with numerous biological properties, and has been commonly used as a folk remedy in Northern European countries for various disorders affecting the digestive system (Shashkina et al., 2006; Balandaykin and Zmitrovich, 2015). The most used formulations are powder, aqueous extract and hydroalcoholic extract, which can be titrated in polysaccharides, beta-glucans, alpha-glucans and polyphenols. *I. obliquus* has also been successfully used to ameliorate the negative effects of DSS in mice, and the polysaccharides of this fungus have shown a positive regulatory effect on the microbiota (Chen et al., 2019). In a model of mice with chronic pancreatitis, the GM profile, compromised by the disease, was partially restored by the administration of *I. obliquus* polysaccharides, which led to increased diversity and richness of GM and also improved the clinical condition of the mouse (Hu et al., 2017). *Ganoderma lucidum* (Reishi in Japanese) is a mushroom with a woody consistency and a bitter taste, which grows preferably on oaks and chestnuts. The main traditional use in China and Japan is aimed at counteracting the allergic and inflammatory state (Bhardwaj et al., 2014). Recent studies have identified more than 400 bioactive molecules present in this mushroom. Some of these were firstly identified in this species and consequently their name referred to the species, such as ganoderiol, ganolucidinic acids and ganoderman-triol (Ahmad, 2018). There is strong evidence of prebiotic activity of *G. lucidum*, although this may be secondary to a direct effect on components of the immune system. In DSS-induced colitis in rats, *G. lucidum* glucans increased SCFA-producing bacteria such as *Ruminococcus*, and reduced pathogens such as *Escherichia* and *Shigella* in both small intestine and cecum (Xie et al., 2019). In high fat diet (HFD)-fed mice, which exhibit body weight gain and diet-associated dysbiosis, treatment with *G. lucidum* mycelium reverted HFD-induced intestinal dysbiosis, decreasing the Firmicutes-Bacteroidetes ratio and the Proteobacteria relative abundance. Furthermore, Reishi treatment reduced metabolic endotoxemia by restoring the integrity of the intestinal barrier. This demonstrated that one of the main mechanisms of action of *G. lucidum* in the intestine is related to the modulation of GM. Polysaccharides with a high molecular weight (>300 kDa) have been identified as the most responsible for this modulation of GM, since these polysaccharides are present in *G. lucidum* in considerable amounts (Chang et al., 2015). Similar results were obtained in a rat model of type 2 diabetes, in which treatment with *G. lucidum* reduced the relative abundance of harmful bacteria, such as *Aerococcus, Ruminococcus, Corynebacterium,* and *Proteus*, while increased that of *Blautia, Dehalobacterium,* and *Parabacteroides*. GM analysis indicated that Reishi treatment could also restore the microbial metabolism of amino acids, carbohydrates, inflammatory substances and nucleic acids, altered by obesity and diabetes (Chen et al., 2020). In a mouse model of pancreatitis induced by diethyldithiocarbamate (DDC), polysaccharides of *G. lucidum* were able to positively modulate the GM, decreasing the relative abundance of Bacteroidetes and increasing that of Firmicutes. Reishi polysaccharide supplementation increased the relative abundance of beneficial bacterial families, such as Lactobacillaceae and Lachnospiraceae, especially *Roseburia*. These results confirmed that the therapeutic mechanism on chronic pancreatitis could also depend on the restoration of a healthy eubiotic GM (Li et al., 2016).

#### Microbiota-Modulating Plants

It should be noted that all plants, if rich in fiber content, can have prebiotic activities. Despite this, some plants in particular have shown the ability to modulate the GM in a much more decisive way than would be expected from their unique prebiotic effect due to their fiber content. *Cichorium intybus* is a perennial herbaceous plant whose rhizome and roots are traditionally used in Europe for the treatment of gastrointestinal disorders (Thumann et al., 2019). Used to supplement the diet of farmed broilers and to improve their production performance, *C. intybus* has been shown to induce significant changes in the ileal microbiota, consisting of lowering *E. coli* counts and increasing *Lactobacillus* ones. These effects have been clearly associated with improved growth performance. Dietary *C. intybus* powder has an undoubted prebiotic effect linked to the high content of soluble fiber, and in particular inulin (Khoobani et al., 2019). However, different modulations of the Firmicutes/Bacteroidetes ratio and of some bacterial genera, such as *Alloprevotella, Blautia, Alistipes* and *Oscillibacter*, were observed in mice fed with different chicory cultivars, with a variable effect depending on the genotype of the chicory and not on the fiber content (Khoobani et al., 2019). *Boswellia serrata* is an arboreal plant that forms an aromatic resin also known as “frankincense”. *B. serrata* resin was used as a supplement in rabbit diets at different dosages to obtain changes in the caecal microbiota. Results indicated that substantial changes were found in the microbial populations in the cecum of rabbits treated with *B. serrata*, with a significant decrease in total bacterial count and in particular a decrease in *Salmonella enteritidis* and *E. coli* compared to the untreated control group. These results could be attributed to the high polyphenol content of *B. serrata* and to the presence of boswellic acids, which have a powerful antimicrobial effect (Ismail et al., 2019). *Pistacia lentiscus* is a shrub or small evergreen tree that produces a resin called Chios mastic gum, used as a natural food supplement. The effect of *P. lentiscus* was studied in mice with obesity, non-alcoholic steatohepatitis (NASH) and HFD-induced liver fibrosis. Treatment with *P. lentiscus* promoted a partial but significant recovery of GM diversity associated with a decrease in the relative abundance of Bacteroidetes (Kannt et al., 2019). *Olea europaea* is an evergreen fruit tree that is traditionally found in the Mediterranean area. The extra virgin oil (EVO) obtained from the fruits of this plant is able to induce a greater GM biodiversity and promote the growth of beneficial commensal bacteria, both in humans and in laboratory animals (Marcelino et al., 2019). The leaf extract of *O. europaea* administered to obese mice was able to improve their GM by partially restoring the quantities of Actinobacteria, Bacteroidetes and Verrumicrobia. Furthermore, the relative abundance of *Akkermansia* spp. was restored, suggesting a possible positive effect on intestinal barrier functions in treated mice (Vezza et al., 2019). As for *Angelica arcangelica, Achillea millefolia* and *Cetraria islandica*, officinal plants traditionally used to treat intestinal dysbiosis and inflammation, there are no scientific studies published so far to support their positive modulation action on GM. This does not mean that these medicinal plants are not effective in modulating GM ecology, but only that documented scientific evidence of their alleged therapeutic activities is still too scarce.

### Essential Oils as Potential Bowel “Eubiotics”

The alteration of the ecologically stable environment of GM can be produced by different causes, such as broad-spectrum antibiotic therapies, xenobiotics in foods, or growth of pathogenic bacterial strains that can interfere in this equilibrium and cause dysbiosis, low-grade gut inflammation or even colitis (Petersen and Round, 2014; Spisni et al., 2020). Thus, GM dysbiosis over time can trigger alterations in the microbial metabolome, increasing the production of toxins that could activate the innate immune response leading to chronic low-grade intestinal inflammation, which could be one of the triggers for the development of several diseases (Kim and Jazwinski, 2018).

Essential oil (EO) molecules are capable of selectively targeting some bacterial species, especially pathobionts, leaving unaltered the bacterial populations considered healthy (Thapa et al., 2012). Furthermore, they are able to counteract the growth of some fungi resident in GM, such as *Candida albicans* whose overgrowth may causes severe opportunistic infections in humans (Saracino et al., 2022). For these reasons these molecules can be considered as eubiotic agents capable of counteracting intestinal dysbiosis. A number of various EO molecules (i.e., eugenol, thymol, piperine) have been used to positively modulate the broiler chicken GM in different studies. The overall results demonstrated that these compounds represent an effective supplementation for the improvement of GM ecology in farmed chickens with a clear eubiotic effect resulting in increased growth performance (Weber et al., 2012).

Geraniol (Ge-OH), an aliphatic terpene alcohol present in EOs extracted from different plants, such as Palmrose (*Cymbopogon martini*), has demonstrated robust anti-dysbiotic activities in mice whose GM ecology was disrupted by DSS administration. Ge-OH, both enema or orally-administered, was able to prevent colitis-associated dysbiosis in treated mice (De Fazio et al., 2016). The eubiotic effect of Ge-OH could be considered multi-target, since this EO compound is a natural inhibitor of the enzyme cyclooxygenase-2 (COX-2), whose activity in the gut wall strongly contributes to intestinal inflammation. Since chronic low-grade inflammation and dysbiosis enter a self-sustaining loop, the multitarget eubiotic action of Ge-OH tends to restore the gut microbial ecology by contrasting both dysbiosis and inflammation. This is probably the reason why the efficacy of Ge-OH delivered directly to the colon reached that of corticosteroid therapy in this model of DSS-induced colitis (De Fazio et al., 2016). In IBS patients, Ge-OH administration led to GM increased biodiversity increased relative abundances of *Collinsella* and especially *Faecalibacterium*, a well-known health-promoting butyrate producer consistently found to be decreased in IBS patients (Rizzello et al., 2018). D-Limonene, administered to HFD mice reduced obesity and reversed many different microbiota signature due to the unbalanced diet, decreasing Peptostreptococcaceae, Desulfovibrionaceae and Erysipelotrichaceae genera in treated mice and increasing the relative abundance of Bacillaceae, Planococcaceae and Clostridiaceae (Valerii et al., 2021). By using an *in vitro* human colon model, D-Limonene was also capable to selectively reduce *Clostridium* group IV (Nissen et al., 2021).

The use of broad-spectrum antibiotics against infectious diseases or to counteract the overgrowth of pathobionts in various diseases affecting the intestine, such as IBS (Andrews et al., 2021) or IBD (Townsend et al., 2019), could be associated with a transient dysbiosis in the gastrointestinal tract (Çalışkan et al., 2022). Thus, a study evaluated the possibility to substitute antibiotic therapy with a mixture containing EOs was performed on piglets. Dietary inclusion of the combination of tributyrin with methyl salicylate and oregano EO was compared to antibiotic treatment in terms of bowel health. The results demonstrated an improved intestinal morphological structure in weaned piglets, with an improved ratio of villus height to crypt depth in their intestine. Moreover, the results showed major changes in the profiles of GM and bacterial metabolites, which were beneficial to the animal health. In conclusion, this study demonstrated that the combination of Tributyrin with methyl salicylate and oregano EO could be a potential alternative to antibiotics as a feed additive in pigs (Zhang et al., 2020).

In 2009, a preliminary *in vitro* study examined the potential of a selection of EOs as agents to treat dysbiosis. These EOs were examined using the agar dilution method and doubling dilutions against 12 intestinal bacteria species, which represent the major taxa found in the human gastrointestinal tract. The best results were obtained using *Carum carvi, Lavandula angustifolia, Trachyspermum copticum,* and *Citrus aurantium* var. amara, so these EOs have been found to be the most promising in the treatment of intestinal dysbiosis, even if more research is needed to investigate the tolerability, safety and selective action of these EOs on other bacterial species (Hawrelak et al., 2009).

A recent study was made to investigate the effects of replacing antibiotics with a combination of plant EOs on the growth performance and gastrointestinal health of broilers. Seven hundred and twenty 1-day-old male broilers were randomly divided into 3 experimental groups: the control treatment, the antibiotic supplementation treatment, or the EO supplementation treatment. The EO supplement consisted of a standard combination of ingredients: eucalyptus EO (25%), carvacrol (35%), cinnamaldehyde (25%), capsaicin (10%), and some other prebiotics (5%). No significant differences were found for food intake, body weight gain, culling rate and carcass performance among the three treatments. Examination of the morphology of the intestinal wall did not show significant differences among treatments. Nevertheless, analysis of the caecal microbiota revealed that only supplementation with combined EOs significantly increased bacterial diversity and some representative probiotic bacteria, particularly *Streptococcus* and *Bifidobacterium* (Xue et al., 2020).

The role of oregano EO as a food supplement has been investigated in farmed animals since this EO, with the major active compounds carvacrol and thymol, has been reported to have antimicrobial and antioxidative properties resulting in improved intestinal barrier function and weight growth in pigs and poultry. However, its impact on GM still remains unclear. Analysis of health records showed that farmed piglets supplemented with oregano EO had a significantly reduced need of therapeutic treatment and overall reduced mortality. In sows and piglets, the GM structure and composition varied considerably over time: sows supplemented with oregano EO during lactation showed an increase in the relative abundance of Lactobacillaceae, Fibrobacteriaceae and Akkermansiaceae, and the analysis of the piglet GM, at two and 4 weeks of age, showed a relative decrease in Enterobacteriaceae and an increase in butyrate producers (from Lachnospiraceae family) at both timepoints. The hypothesis to explain these findings was that this GM modulation in piglets was dependent on maternal microbial transfer (Hall et al., 2021).

Table 2summarize the major effects of fungus, plants and EO molecules on the GM modulation in humans and animal models of diseases.

**TABLE 2:** Fungus, plants and EO molecules as GM modulators in clinical studies and in preclinical model of non-communicable diseases.

Diseases/Therapies	Model	Main findings
Gastrointestinal disorders		
Colitis		
*Hericium erinaceus* (HEP3 protein)	Rats, TNBS; Rats, acetic acid	Overall reversal of colitis-associated dysbiosis (rise of Actinobacteria*,* Tenericutes*,* SCFA producers, decrease of Bacteroidetes and Firmicutes)
*Hericium erinaceus* (dry extract	Mice, DSS	Overall reversal of DSS-induced dysbiosis (decrease of *Akkermansia muciniphila*, increase of SCFA producers)
*Ganoderma lucidum* (glucans)	Mice, DSS	Overall reversal of DSS-induced dysbiosis (rise of *Ruminococcus*, decrease of *Escherichia* and *Shigella*)
Geraniol	Mice, DSS	Overall reversal of DSS-induced dysbiosis (increase of Lactobacillaceae*,* Bacillaceae and Bacteroidetes)
IBS		
Geraniol	Human	Overall reversal of IBS-associated dysbiosis (increase of *Collinsella* and *Faecalibacterium*)
Pancreatitis		
*Inonotus obliquus* (polysaccharides)	Mice, DDC	Overall reversal of DDC-associated dysbiosis (increase of Bacteroidetes, decrease of Firmicutes)
*G. lucidum* (polysaccharides)	Mice, DDC	Overall reversal of DDC-associated dysbiosis (decrease of Bacteroidetes and increase of Firmicutes, Lactobacillaceae*,* Lachnospiraceae, *Roseburia*)
Metabolic Disorders		
Obesity		
*G. lucidum* (mycelium	Mice, HFD	Overall reversal of HFD-induced dysbiosis (decrease of the Firmicutes*-*Bacteroidetes ratio, and Proteobacteria)
*Pistacia lentiscus*	Mice, HFD	Reversal of HFD-induced dysbiosis (partial recovery of diversity, decrease of Bacteroidetes)
*Olea europaea*	Mice, HFD	Reversal of HFD-induced dysbiosis (rise of Actinobacteria*,* Bacteroidetes, Verrucomicrobia, *Akkermansia* spp.)
D-Limonene	Mice, HFD	Reversal of HFD-induced dysbiosis: increase of Bacillaceae, Planococcaceae, Clostridiaceae; decrease of Peptostreptococcaceae, Desulfovibrionaceae, Erysipelotrichaceae
T2D		
*G.lucidum* (Glucans)	Rat, HFD and streptozotocin	Overall reversal of T2D-associated dysbiosis (decrease of *Aerococcus, Ruminococcus, Corynebacterium* and *Proteus*, increase of *Blautia, Dehalobacterium, Parabacteroides*)
Disease-Unrelated Eubiotic Properties		
*Cichorium intybus*	Farmed broilers	Significant changes in the ileal microbiota (lower *Escherichia coli,* rise of *Lactobacillus*)
*C. intybus*	Mice	Lowering of the Firmicutes*/*Bacteroidetes ratio, increased *Alloprevotella, decreased Blautia, Alistipes* and *Oscillibacter*
*Boswellia serrata* (resin)	Rabbit	Decrease of bacterial counts, *Salmonella enteritidis* and *E. coli*
Oregano EO (in combination with trybutirin and methyl salicylate)	Piglets	Increase of Firmicutes, decrease of Proteobacteria, Actinobacillus, *Escherichia*

## Conclusion and Prospective

There is no doubt that GM modulation represents a therapeutic frontier for the prevention and treatment of many different diseases. Probiotics are the most widely used type of supplement to date for this purpose, even if not always supported by scientific clinical data. On the other hand, the correct intake of prebiotics can be easily achieved with a diet that includes a large consumption of vegetables, but their supplementation requires further studies that define which types and which dosages. Postbiotics have so far shown more limitations than real therapeutic successes. The potential role of fungi and medicinal plants in GM modulation has been tested and analyzed only for a few of them and mainly on animal models. EOs or their single components are widely used in animal breeding in an attempt to reduce the use of antibiotics in meat farms. Their eubiotic effect on GM is demonstrated and, at the doses used, no side effects related to their toxicity appear to be evident. They are certainly able to positively modulate the human GM as well, selectively acting on pathobionts, without altering or even improving the fraction of health-associated commensals. However, new human clinical studies on EO or their single compounds are needed to verify the possibility of being specifically used in the context of metabolic pathologies or diseases in which dysbiosis plays a key role in the pathogenesis.

## Data Availability

The datasets presented in this study can be found in online repositories. The names of the repository/repositories and accession number(s) can be found below: Original raw paired-end sequence data are available in NCBI data base with BioProject accession number PRJNA795336.
